# Knowledge and Perceptions of Blood Safety among Blood Donors in Kelantan, Malaysia

**DOI:** 10.21315/mjms2019.26.6.13

**Published:** 2019-12-30

**Authors:** Pei Pei Tan, Hafizuddin Mohamed Fauzi, Rosnah Bahar, Chee Tao Chang, Nur Arzuar Abdul Rahim

**Affiliations:** 1 Regenerative Medicine Cluster, Advanced Medical and Dental Institute, Universiti Sains Malaysia, Kepala Batas, Pulau Pinang, Malaysia; 2 Department of Haematology, School of Medical Sciences, Universiti Sains Malaysia, Kubang Kerian, Kelantan, Malaysia; 3 Clinical Research Centre, Hospital Raja Permaisuri Bainun, Ministry of Health Malaysia

**Keywords:** blood safety, blood donors, knowledge, perception, blood transfusion

## Abstract

**Background:**

Unsafe blood products may cause transfusion-transmissible infections. This study aimed to evaluate the knowledge and perceptions of blood donors regarding blood safety.

**Methods:**

This was a cross-sectional study conducted in the Kelantan state of Malaysia. The questionnaire comprised 39 questions that covered areas such as donors’ social demographic information, knowledge of transfusion-transmitted diseases, blood screening and donor eligibility and perceptions towards blood safety. The knowledge score was categorised as good or poor.

**Results:**

Of the 450 distributed questionnaires, 389 were suitable for analysis. Only 18.5% of the donors had good knowledge, with 81.5% having poor knowledge. Less than 30% were aware that people with multiple sexual partners, bisexual people and male homosexual people are permanently deferred from blood donation. Only 29.4% agreed that donors are responsible if their blood causes infection. Furthermore, 39.3% assumed that they could check their HIV status through blood donation, and 10.3% and 5.4% of the respondents believed that donors are free from infection if they wear a condom during sex or only have oral sex when involved in prostitution, respectively.

**Conclusion:**

Poor knowledge and notable misperceptions concerning safe blood donation were found among blood donors. The Ministry of Health should incorporate safe blood education in future public awareness programmes.

## Introduction

Blood safety is defined as the degree to which the blood supply for blood transfusions is free of harmful substances or infectious agents and correctly typed and cross-matched to ensure serological compatibility between blood donors and recipients (1). Neglecting blood safety may cause blood products to carry transfusion-transmissible infections (TTIs). The World Health Organization (WHO) guidelines recommend that several routine laboratory tests be performed to donated blood for potential TTIs, including human immunodeficiency virus (HIV), hepatitis B virus (HBV), hepatitis C virus (HCV) and syphilis (2). Hence, screening tests are implemented in most countries to reduce the occurrence of TTIs.

Despite the screening tests performed, unsafe blood products remain prevalent in developing countries (3). An estimated 5%–15% of HIV infections in developing countries are caused by unsafe blood transfusions (4). The cost of unsafe blood is immense, causing a loss of productive labour as well as an increased burden on healthcare systems. One of the reasons for TTIs is the donation of blood from donors with high-risk behaviours (5). As a preventive measure, blood donor screening questionnaires had been developed to assess the health status of donors and their suitability for donation. This also helps exclude subjects at high risk of transmitting blood-borne infectious pathogens through blood donation (6).

Blood donor questionnaires rely mainly on the cooperation of blood donors in providing the information related to their health status and risk of exposure to infections (3). Not all blood donors disclose deferrable risk behaviours during blood donation as the questionnaires contain socially sensitive questions that seek responses regarding sexual experience and the use of illicit drugs. A study in Hong Kong reported that 10.2% of the donors who had donated blood possibly had deferrable behaviours that were not disclosed prior to blood donation (7). The importance of research on behavioural risk factors among blood donors, particularly in developing countries, has therefore been emphasised over the years (4).

Blood safety knowledge levels vary among developing countries. Researchers have found that, in Southern Ethiopia, only 38% of the population have adequate knowledge (8). Important information, such as the association of TTIs with permanent deferrable risk behaviours (multiple sexual partners, male-to-male sex and intravenous drug abuse) and temporary deferrable risks (tattoos or acupuncture in the past six months, tooth extraction in the past 24 h and staying with family members who have HBV or HCV), has not previously been assessed among blood donors (9). A study in the United States reported that 23% of the respondents thought that it was appropriate to donate blood in order to be tested for HIV (10). In another study in Serbia, 2.8% of the blood donors either strongly disagreed or disagreed that truthful and accurate answers to the questions on donor questionnaires were essential for the safety of patients who received that blood (11).

Kelantan is a state in Malaysia that located in the north of the country and borders Thailand. A local study conducted in Kelantan determined that the prevalence of HBV infection among blood donors was 1.1% (12). Another study revealed a 0.14% prevalence of HCV infection among blood donors in the north-eastern region of Malaysia (13). These two studies highlighted that improvements in knowledge and the availability of information on HBV and HCV infection are needed among the public to lower the incidence of TTIs. A study conducted at the National Blood Centre of Malaysia reported that the seroconversion rates of syphilis, HIV and HCV increased notably from 2004 to 2008 (14). Despite this alarming scenario, there have been no local studies to explore blood donor knowledge and perceptions regarding blood safety. This study therefore aimed to provide insights regarding the knowledge and perceptions of blood donors on blood safety as this may be useful for the development of donor educational materials and encourage the practice of self-deferral among blood donors.

## Methods

This was a cross-sectional study conducted in the Kelantan state of Malaysia. The study was conducted from June 2018 to May 2019 using a self-administered structured validated questionnaire developed in the Malay language, which is the national language of Malaysia. All blood donors who were older than 18 years of age, understood the Malay language and were eligible to donate or were temporarily deferred from donation were invited to participate in the study. Donors who were illiterate, non-Malaysian, medical personnel or health sciences students and/or had any known mental disorder were excluded.

The questionnaire underwent face and content validation by a panel of 10 multidisciplinary experts and two transfusion medicine specialists. Subsequently, a pilot test was conducted among 130 blood donors at the National Blood Centre. The test–retest method was employed whereby the participants were tested twice with the same set of questionnaires. An intraclass correlation coefficient (ICC) of between 0.4 and 0.75 was considered acceptable, and an ICC value of ≥ 0.75 was considered excellent. The reliability of the questionnaire was confirmed, with an ICC value of > 0.8 for the knowledge domain and > 0.6 for the perception domain (15).

The questionnaire comprised 39 questions, which were divided into four sections: (i) social demographic information (10 items), (ii) knowledge of transfusion-transmitted diseases and blood screening (10 items), (iii) knowledge of donor eligibility (9 items) and (iv) perceptions of donors towards blood safety (10 items). The knowledge score was further categorised according to an arbitrary cut-off point, where good was 60%–100% (12–19 marks) and poor was ≤ 59% (0–11 marks). The perception items were not summated but instead classified individually as inappropriate or appropriate.

The sample size was calculated based on 5% precision and a 95% confidence level with an infinite population using a single proportion calculation where 38.3% of the population had adequate knowledge (8). A minimum sample size of 399 was required. The questionnaires were distributed to potential respondents by the researchers at 16 mobile sites in the Kelantan state. Systematic random sampling was employed in which every second registered blood donor was recruited. The respondents who consented in writing were given the self-administered questionnaire, which took about 20 min to complete. The questionnaires were returned to the researchers on the same day.

Statistical analysis was performed using SPSS version 22.0 for Windows (SPSS, Chicago, IL). The sociodemographic data, knowledge and perceptions of the blood donors were analysed and presented descriptively. The categorical data were expressed as frequency (percentage) and the numerical data as mean (SD). Logistics regression was used to determine the association between the sociodemographic characteristics and the outcomes. A *P*-value of less than 0.05 was considered statistically significant.

Ethical clearance was obtained from the Human Research Ethics Committee of Universiti Sains Malaysia and the Medical Research and Ethics Committee of the Ministry of Health, Malaysia. All the questionnaires were anonymous. The data were presented as grouped data and did not identify the respondents individually. All the written research documents, including the study data (demographic and clinical data), were protected by the researchers. The investigators declared no conflicts of interest.

## Results

Of the 450 distributed questionnaires, 437 sets of questionnaires were returned, giving a response rate of 97.1%. Questionnaires with missing data were excluded, so 389 complete questionnaires were analysed in this study. The mean age of the respondents was 25.79±8.40 years. Most of the respondents were Malay (92.3%), single (72.2%), students (52.4%) and regular donors (56.3%) and had a bachelor’s degree or above (43.4%) (Table 1). Of the 389 respondents, 72 (18.5%) had good knowledge scores, but most of them (81.5%) had poor knowledge scores.

Two hundred and fifty-two (64.8%) respondents were aware that infection could occur through a blood transfusion. Less than one third knew that dengue (33.2%), Zika (32.9%) and mad cow disease (18.0%) can be contracted through a blood transfusion. Only 73.0% (*n* = 284) answered correctly that an HIV screening test was done for all donated blood. Two thirds or less of the respondents knew that all donated blood was screened for HBV (66.8%, *n* = 260), HCV (59.1%, *n* = 230) and syphilis (57.3%, *n* = 223).

Less than 30% of the respondents were aware that people with multiple sexual partners, bisexual people and male homosexual people are permanently deferred from blood donation (Table 2), and only 29.4% of the respondents strongly agreed or agreed that donors are responsible if their blood causes infection in a recipient. Furthermore, 39.3% of the donors strongly agreed or agreed that they could check their HIV status through blood donation, and 10.3% and 5.4% of the respondents strongly agreed or agreed that donors are free from infection if they wear a condom during sex or only have oral sex when involved in prostitution (Table 3).

The respondents with a secondary or lower educational level had 4.1 times higher odds of poor knowledge compared to those with a bachelor’s degree or above (*P* = 0.010). Those with a low or very low household income had 2.8 times (*P* = 0.010) and 2.3 times (*P* = 0.014) higher odds of poor knowledge, respectively, on blood safety compared to those with a high household income (Table 4).

## Discussion

The majority of the respondents in our study obtained a poor knowledge score although more than half of the respondents were regular blood donors and had received a tertiary education. Most of the respondents were unable to identify the diseases that could be contracted after an erroneous blood transfusion and were unaware of the high-risk behaviours and practices that may lead to permanent or temporary blood donation deferral.

Overall, more than 80% of the respondents in this study had poor knowledge. This is consistent with a study conducted in India which showed that only 33.1% of the respondents had adequate safe blood donation knowledge (16). The findings of our study may be useful to healthcare providers and stakeholders as recognising the low levels of knowledge of blood donors in local communities could help in the planning of feasible strategies to improve public knowledge on blood safety and self-deferral.

Our study findings revealed poor knowledge among blood donors regarding TTIs: less than one third of the donors were aware that dengue, Zika and mad cow disease could be transmitted through a blood transfusion. Dengue fever is endemic in Malaysia, with more than 60,000 cases reported from January to June 2019. In 2015, there were laboratory-confirmed cases of Zika virus infection among travellers returning from Malaysia, for whom the Zika virus was probably associated with microcephaly (17, 18). It is therefore important to raise awareness about TTIs.

In this study, less than 75% of the respondents were aware that all donated blood is screened for HIV, and less than two thirds were aware that donated blood is screened for HCV and syphilis. A similar study conducted in the United States found that slightly less than 80% of the respondents knew that donated blood was tested for HIV (19). According to the WHO guidelines, all blood should be routinely screened for HIV, HBV, HCV and syphilis (20).

More than half the respondents did not know that invasive procedures such as aesthetic injections, cupping *(bekam)*, acupuncture, body piercing and tattoos may lead to a temporary deferral of blood donation. Furthermore, it is noteworthy that less than one third of the respondents were aware that high-risk behaviours such as multiple sexual partners, male homosexual relationships, bisexual relationships and illegal intravenous drug usage would lead to a permanent deferral of blood donations. In a study conducted in Norway, approximately 10% of 127 repeat donors were denied the opportunity to donate blood due to drug abuse, while 2% of the repeat donors were permanently deferred as they were in male homosexual relationships (21). Another study in Japan reported that 23 of 5,585 blood donors were deferred due to unsafe sexual practices or drug abuse (22). Face-to-face donor interviews may not ensure truthful declarations by donors (23); however, by instilling donors with good knowledge about the permanent and temporary donor deferral criteria, it is hoped that voluntary self-deferral among blood donors will be possible.

In our study, knowledge score was significantly associated with educational level and household income. Higher socioeconomic status is commonly considered a predictor of better knowledge (24, 25). A British cohort study reported that the study participants who came from a lower socioeconomic background demonstrated significantly poorer academic performance than those higher in the social hierarchy (26). Fair and equal access to health information is important to reduce the gaps in knowledge between those who come from distinct socioeconomic backgrounds (25). It is therefore imperative that education on blood safety for those from lower socioeconomic backgrounds be prioritised.

It was alarming to note that 39.3% of the respondents in our study strongly agreed or agreed that donors could donate blood to check their HIV status. Similar to our findings, another study reported that more than one third of blood donors felt that it was acceptable to donate blood to screen for HIV (19). Action should be taken to prevent such behaviours, for example, by incorporating the impact of transfusion-transmittable diseases into the current school education syllabus.

Not all blood donors reveal their high-risk behaviours during blood donation (7, 23, 27), yet false declarations may lead to TTIs among recipients. Our study found that 7.2% of the respondents strongly disagreed or disagreed that donors who give untruthful declarations should be brought to justice. A study conducted in Serbia reported that 2.8% of the study participants disagreed that they should give truthful answers in the donor questionnaire (11). In view of the potentially catastrophic outcomes associated with such practices, drastic measures, such as enacting laws to restrict false blood donation declarations, may be warranted by the government.

In addition, 51.2% of the donors in our study falsely believed that their blood is perfectly safe if the screening test results are negative. In a study conducted among American blood donors, 40% of the donors did not know that the available screening tests may not detect a recent infection (10). In a 2012 study conducted by Steele et al. (19), 17.5% of the respondents were not aware of the ‘window period’ of the HIV screening test. The local donors in our study were similarly ignorant of the screening test window period. There is thus a clear urgency to improve donors’ awareness regarding the limitations of the screening tests undertaken after blood donation and the implications of high-risk behaviours.

In terms of perceptions about sexual behaviour, 10.3% and 5.4% of the respondents in our study strongly agreed or agreed that blood is safe from infection if donors wear a condom during sex or only have oral sex during prostitution, respectively. This was a major misconception among the blood donors who thought that common preventive measures can completely prevent them from contracting sexually transmissible infections (STIs). Previous studies have found that oral sex may cause STIs while condom failure is not uncommon (28, 29). Accordingly, awareness regarding STIs should be enhanced to ensure transfusion blood safety.

To the best of our knowledge, this is the first local study to assess the knowledge and perceptions of blood donors concerning safe blood donation. The study questionnaires were distributed to a wide range of blood donors at multiple donor sites in the Kelantan state, which suggests the generalisability of the findings. However, a few limitations were also identified. This study may not represent the knowledge and perception levels among donors in other states of Malaysia where people have different socioeconomic backgrounds. Since the study only included blood donors, the knowledge and perceptions of the general public were not represented. In future, a large-scale nationwide study that includes the general public should be undertaken to assess blood safety knowledge among the general public.

## Conclusion

The blood donors in this study had poor knowledge and notable misperceptions regarding what constitutes safe blood. The study findings can be utilised by the Ministry of Health of Malaysia to guide further planning of safe blood educational programmes aimed at the public, to raise awareness among blood donors and to reduce the incidence of untruthful blood donation declarations.

## Figures and Tables

**Table 1:** Demographic characteristics of donors

Characteristics	Frequency (*n*)	Percentage
Age^a^ (years)	25.79±8.40	
Gender		
Male	186	47.8
Female	203	52.2
Race		
Malay	359	92.3
Chinese	20	5.1
Indian	7	1.8
Others	3	0.8
Marital status		
Single	281	72.2
Married	104	26.7
Divorced	1	0.3
Widow	3	0.8
Household income^b^ (RM)	Median: 2500 (IQR: 3700)	
High income	0	0.0
Medium income	55	14.1
Low income	101	26.0
Very low income	233	59.9
Occupation		
Government	42	10.8
Private	118	30.4
Self-employed	20	5.1
Student	204	52.4
Unemployed	5	1.3
Education level		
Degree/master/PhD	169	43.4
Diploma	159	40.9
Secondary school	60	15.4
No formal education	1	0.3
Donation status		
First time donor	124	31.9
Regular donor	219	56.3
Lapsed donor	46	11.8

Notes:

a Age is expressed as mean standard deviation

b Household income is expressed in both median (interquartile range) and categories. Income was categorised according to the Report of Household Income and Basic Amenities Survey 2016. High income: > RM13,146 Medium income: RM6,275–RM13,146, Low income: RM3,000–RM6,274 Very low income: < RM3,000 (30)

**Table 2:** Responses on eligibility of blood donation

Responses on eligibility criteria of blood donation	Correct respondents (*n*)	Correct percentage (%)
1. Frequent change of sexual partner	109	28.0
2. Male homosexual	112	28.8
3. Bisexual	102	26.2
4. Aesthetic injection on day of donation	182	46.8
5. Intravenous drug user	146	37.5
6. Cupping	196	50.4
7. Acupuncture	146	37.5
8. Body piercing	149	38.3
9. Tattoo	80	20.6

**Table 3:** Perception of donors towards blood safety

Perception statement	Strongly disagree (%)	Disagree (%)	Unsure (%)	Agree (%)	Strongly agree (%)
1. Donors are responsible if their blood causes infection to the patient	8.7	34.4	27.5	21.9	7.5
2. Someone who is having fever can donate blood	23.7	47.6	19.8	8.2	0.8
3. Donors can donate blood to check the HIV status of themselves	20.3	16.5	23.9	32.1	7.2
4. Donors should not donate blood if they were aware that their blood is unsafe to be given to the patients	3.1	1.5	7.5	32.6	55.3
5. Blood donors who gave untruthful declaration should be brought to justice	2.3	4.9	21.1	41.1	30.6
6. Donors’ blood is 100% safe if the screening test is negative	5.4	5.7	37.8	31.1	20.1
7. The blood of the donors are free from infection and can be donated if:					
(a) Donors wear condom when involved in prostitution or having multiple sexual partners	22.1	24.2	43.4	8.5	1.8
(b) Donors use the same spoon when eating with HIV patients	20.3	26.2	37.5	14.1	1.8
(c) Donors stay in the same house with Hepatitis B patient	9.8	22.6	53.0	12.9	1.8
(d) Donors involved in oral sex only when getting the service of prostitute	25.4	22.4	46.8	4.6	0.8

**Table 4:** Association of knowledge level with respondents’ sociodemographic background

Variable	Simple logistic regression			Multiple logistic regression*		
	
	b	OR (95% CI)	*P*	b	OR (95% CI)	*P*
Age, years	0.028	1.028 (0.994, 1.064)	0.107	-	-	-
Gender						
Male	0	1		-	-	-
Female	−0.517	0.596 (0.353, 1.008)	0.054			
Ethnicity						
Malay	0	1	0.781	-	-	-
Chinese	−0.086	0.918 (0.297, 2.834)	0.881			
Indians/Others	0.725	2.065 (0.257, 16.579)	0.495			
Education level						
Degree and above	0	1	0.013	0		
Diploma	0.441	1.554 (0.903, 2.676)	0.112	0.366	1.442 (0.830, 2.506)	0.194
Secondary and below	1.518	4.564 (1.561, 13.348)	0.006	1.420	4.139 (1.400, 12.236)	0.010
Household income						
Middle income	0	1	0.006	0		
Low income	1.107	3.026 (1.386, 6.606)	0.005	1.042	2.836 (1.287, 6.248)	0.010
Very low income	0.996	2.708 (1.406, 5.217)	0.003	0.840	2.316 (1.187, 4.518)	0.014
Marital Status						
Single	0	1	0.428	-	-	-
Married	0.412	1.510 (0.813, 2.805)	0.192			
Divorced/widow/others	19.834	411080657.5 (0.000, –)	0.999			
Donor type						
New donor	0	1		-	-	-
Repeat donor	−0.607	0.545 (0.299, 0.995)	0.048			
Donor status						
New donor		1	0.136	-	-	-
Regular donor	−0.557	0.573 (0.308, 1.064)	0.078			
Lapsed donor	−0.752	0.471 (0.200, 1.111)	0.085			

Notes: b = regression coefficient, OR = odds ratio, CI = confidence interval.

* Forward stepwise multiple logistic regression analysis. Multicollinearity and interaction term were checked and not found. Hosmer-Lemeshow test (*P* = 0.988), classification table (overall correctly classified percentage = 81.4%) and area under curve (64.5%) were applied to check model fitness.

## References

[1] National Center for Biotechnology Information (2011). Blood Safety–MeSH–NCBI.

[2] World Health Organization (2009). Screening donated blood for WHO transfusion-transmissible infections.

[3] Lin C, Leung J, So B, Lee C (2014). Donor selection for blood safety: is it still necessary?. ISBT Sci Ser.

[4] World Health Organization (2009). Patient Safety Research.

[5] Dhingra D (2002). Blood safety in the developing world and WHO initiatives. Vox Sang.

[6] Tagny C, Kouao M, Touré H, Gargouri J, Fazul A, Ouattara S (2011). Transfusion safety in francophone African countries: an analysis of strategies for the medical selection of blood donors. Transfusion.

[7] Wong H, Lee S, Lee C, Chan D (2015). Failure of self-disclosure of deferrable risk behaviors associated with transfusion-transmissible infections in blood donors. Transfusion.

[8] Addisu AG, Sultan HS, Deginet T, Addishwot Z, Samuel T, Bereket A (2017). Assessment of knowledge, attitude and practice of voluntary blood donation and associated factors among residents of Birbir town. J Community Med Health Educ.

[9] World Health Organization (2012). Blood donor selection: guidelines on assessing donor suitability for blood donation.

[10] Sharma U, Schreiber G, Glynn S, Nass C, Higgins M, Tu Y (2001). Knowledge of HIV/AIDS transmission and screening in United States blood donors. Transfusion.

[11] Bogdanović S, Đurić P, Jovanović R, Bogdanović J (2017). Blood donors’ awareness and attitudes towards blood transfusion safety in the autonomous province of Vojvodina, Serbia. Transfusion Medicine.

[12] Yousuf R, Rapiaah M, Ahmed SA, Rosline H, Salam A, Selamah S (2007). Trends in hepatitis B virus infection among blood donors in Kelantan, Malaysia: a retrospective study. Southeast Asian J Trop Med Public Health.

[13] Haslina MN, Khairiah Y, Zainy DZ, Shafini MY, Rosnah B, Marini R (2012). Seroprevalence of hepatitis C virus infection among blood donors in a teaching hospital in northeastern Malaysia. Southeast Asian J Trop Med Public Health.

[14] Nafishah A, Asiah MN, Syimah AT, Mohd Zahari T, Yasmin A, Normi M (2014). Rate of seroconversion in repeat blood donors at the National Blood Centre, Kuala Lumpur. Indian J Hematol Blood Transfus.

[15] Rosner B (2006). Fundamentals of biostatistics.

[16] Sanayaima HD, Jalina L, Shantibala K, Vijaya E (2012). Knowledge, Attitude and Practice (KAP) of blood safety and donation. Indian Medical Gazette.

[17] World Health Organization (2019). Zika epidemiology update.

[18] World Health Organization (2019). Dengue situation update number 572.

[19] Steele W, High P, Schreiber G (2012). AIDS knowledge and beliefs related to blood donation in US adults: results from a national telephone survey (CME). Transfusion.

[20] World Health Organization (2019). Screening donated blood for WHO transfusion-transmissible infections.

[21] Reikvam H, Svendheim K, Røsvik A, Hervig T (2012). Questionnaire-related deferrals in regular blood donors in Norway. J Blood Transfus.

[22] Ngoma A, Goto A, Nollet K, Sawamura Y, Ohto H, Yasumura S (2014). Blood donor deferral among students in northern Japan: challenges ahead. Transfusion Medicine and Hemotherapy.

[23] Fielding R, Lam T, Hedley A (2006). Risk-behavior reporting by blood donors with an automated telephone system. Transfusion.

[24] Feinberg I, Frijters J, Johnson-Lawrence V, Greenberg D, Nightingale E, Moodie C (2016). Examining associations between health information seeking behavior and adult education status in the US: an analysis of the 2012 PIAAC data. PLoS One.

[25] Thiele T, Singleton A, Pope D, Stanistreet D (2014). Predicting students’ academic performance based on school and socio-demographic characteristics. Studies in Higher Education.

[26] Feinstein L (2003). Inequality in the early cognitive development of British children in the 1970 cohort. Economica.

[27] O’Brien S, Xi G, Yi Q, Goldman M (2010). Understanding non-disclosure of deferrable risk: a study of blood donors with a history of intravenous drug use. Transfusion Medicine.

[28] Edwards S, Carne C (1998). Oral sex and transmission of non-viral STIs. Sex Transm Infect.

[29] Bradburn C, Wanje G, Pfeiffer J, Jaoko W, Kurth A, McClelland R (2016). Risky business: condom failures as experienced by female sex workers in Mombasa, Kenya. Cult Health Sex.

[30] Department of Statistics Malaysia (2019). Report of household income and basic amenities survey 2016.

